# 

**Published:** 1887-10-08

**Authors:** 


					u- J
{ No. 54, Vol. III.
October 8th, 1887.
PRICE O'NE PENNY. Q
u fc* Per Annum, 6s. 6d.,post free.
rtt . \1\ Ml Jffl /fiHl Monthly Parts, 6d.; post free, 7id.
[Registered at the General PostOfflce V?\ Jml   Ditto nl)1. i?? <?o /a *. I
** a Newspaper.] 0 P?r Ann" 7s- 6d ? Post free*
'rimix ItfJiirhw, anfr l^ljilantljnrpn,

				

